# Effectiveness of home safety training and balance exercises in reducing fear of falling among older women: A quasi-experimental study in southern Iran

**DOI:** 10.1016/j.pmedr.2025.103351

**Published:** 2025-12-15

**Authors:** Saiedeh Nazari, Fakhruddin Boraghi, Masoud Karimi, Mojtaba Kamalinia, Samuel T. Faloye, Abdolrahim Asadollahi

**Affiliations:** a Dept. of Gerontology, School of Health, Shiraz University of Medical Sciences, Shiraz, Iran; b School of Health, Student Research Committee, Shiraz University of Medical Sciences, Shiraz, Iran; c Dept. of Health Promotion, School of Health, Shiraz University of Medical Sciences, Razi Ave., Shiraz, Iran; d Dept. of Occupational Health and Safety Engineering, School of Health, Shiraz University of Medical Sciences, Shiraz, Iran; e Dept. of Information Systems and Technology, University of KwaZulu-Natal, King Edward Ave., Scottsville, Pietermaritzburg, P.O. Box: 3201, South Africa; f Dept. of Gerontology, School of Health, Shiraz University of Medical Sciences, Razi Ave., Shiraz, Iran

**Keywords:** Accidental falls, Aged, Balance exercises, Ergonomics, Exercise therapy, Fear of falling, Home ergonomic indicators, Home safety, Human, Iran, Older women, Quasi-experimental studies, Fear, Female

## Abstract

**Objectives:**

This study examined whether a combined home-safety training and balance-exercise program could reduce fear of falling and improve ergonomic risks.

**Methods:**

This quasi-experimental study included 336 Iranian women aged ≥60 who were non-randomly assigned to four groups based on the pre-existing type of anti-slip flooring in their homes. Over a two-month period in 2024, participants received home-safety training focused on practical ergonomic modifications, along with supervised balance and strength exercises. Outcomes included the Fear of Falling questionnaire, Berg Balance Scale, Timed Up and Go test, and an ergonomic home-risk checklist.

**Results:**

The intervention significantly reduced fear of falling and improved home ergonomic safety (*p* = 0.01). No significant changes were observed in balance performance (Berg: *p* = 0.82; TUG: *p* = 0.34) or fall frequency (*p* = 0.46). Among flooring types, the Sonia group showed the largest reduction in fear of falling (*p* = 0.02), while the Selda group showed a small decrease in fall frequency (p = 0.01). Ergonomic improvements were most evident in bathrooms and kitchens, where grab bars and non-slip surfaces were implemented.

**Conclusions:**

Home safety training combined with targeted ergonomic changes effectively reduced fear of falling and improved home safety.

## Highlights

• Home-safety training plus balance exercises cut fear of falling by 34 %.
• The intervention improved home safety and led to key home modifications.
• No significant overall gains in balance or fall frequency were observed.
• Sonia flooring showed the largest drop in fear of falling; Selda slightly cut falls.
• Longer interventions may be needed to improve balance and reduce falls.

## 1 Background

Falls are a major health issue in older adults, leading to reduced quality of life, disability, and high healthcare costs (Stavropoulos et al., 2020). Globally, over 37 million falls require medical care each year, with adults over 60 experiencing most fatal events; low- and middle-income countries account for more than 80 % of fall-related deaths (World Health Organization, 2021). Common contributors include chronic diseases, inactivity, high BMI, musculoskeletal problems, and environmental hazards such as slippery floors, poor lighting, clutter, and unsafe bathrooms (Yosef et al., 2024). Fear of falling further impairs balance and restricts daily activities (MacKay et al., 2021; Liu et al., 2021). Home-environment factors—particularly flooring quality—play a central role in fall risk, yet the comparative effects of different flooring types (Sonia, Lotus, Aphrodite, Selda) remain understudied (Boonkhao et al., 2024).

Balance and strength exercises are effective fall-prevention strategies (Stanghelle et al., 2020), but their integration with ergonomic home modifications has been insufficiently evaluated. Older women were the target group due to their higher exposure to household hazards, traditional caregiving roles, and greater physiological vulnerability, including higher osteoporosis prevalence and musculoskeletal weakness (Sarkar et al., 2023; Parab et al., 2024; Sebastiani et al., 2024). Their longer life expectancy also prolongs exposure to risk factors (Özer et al., 2023).

The study was conducted in southern Iran, where hot, dry weather keeps older adults indoors for long periods. Indoor exposure, frequent floor washing, and tiled wet areas (bathrooms, kitchens) may heighten fall hazards. This study therefore examined whether a combined program of home-safety training and balance exercises could reduce fear of falling, improve home ergonomics, and lower fall risk among older women.

## 2 Materials and methods

### 2.1 Study design and population

This quasi-experimental one-group before–after study included adults aged ≥60 with normal baseline balance and no history of severe falls. Conducted at Older Adults Day Care Centers in Shiraz [29°35′30″N, 52°35′01″E], southern Iran, the study assessed a two-month intervention combining home-environment modification training and supervised balance exercises (mid-April to late July 2024). The design is considered quasi-experimental because participants were not randomly assigned to conditions; instead, all eligible older adults attending the centers during the recruitment period were consecutively enrolled into a single intervention group and evaluated before and after the program. No control group was used due to ethical and operational constraints—specifically, withholding a multifaceted fall-prevention intervention from high-risk older adults was deemed unacceptable by the centers. This approach maximized participation but limits causal inference, as changes cannot be fully attributed to the intervention. Future research should incorporate randomized or controlled designs to strengthen internal validity.

### 2.2 Participants

The study population included older adults registered at day care centers in Shiraz, Iran. From an initial list of 478 eligible participants, 336 women aged 60–74 were selected via systematic random sampling and divided into four groups (84 each) based on their home bathroom and toilet flooring type: Sonia, Lotus, Aphrodite, or Selda. These four types were identified as the most common anti-slip floorings through a one-month market survey in February 2024. All groups received the same combined intervention of home safety training and supervised exercises. Sample size was calculated using NCSS PASS 15, with 99 % confidence, 95 % power, α = 0.01, effect size = 0.93, and SD = 0.33 from Delbaere et al. (Delbaere et al., 2021), accounting for a 10 % dropout rate. Inclusion criteria were: no history of severe falls, normal TUG results, Fear of Falling score ≥ 20, and no use of walking aids. Exclusion criteria included new musculoskeletal problems, cognitive impairment, withdrawal, or death. No control group was included, as all participants received the intervention.

### 2.3 Measures

To assess eligibility, demographic questionnaires, the Fear of Falling (FoF) questionnaire, the Timed Up and Go (TUG) test, the Berg Balance Scale-9 (BBS-9) test, and a home ergonomic risk factor checklist were administered. Individuals who scored within the normal range for fall risk were included in the intervention. Data collection tools included a demographic questionnaire (see Supplementary Table 1 for the complete list of items and response categories), the FES-I questionnaire (short version with seven questions for assessing fear of falling), the BBS-9 test (for static balance assessment), the TUG test (for dynamic balance and mobility evaluation), and a home ergonomic risk factor checklist.

The home ergonomic risk factor checklist comprised 96 dichotomous (yes/no) items designed to identify specific environmental fall hazards (‘risk factors’) across key areas of the home, such as bathrooms (e.g., absence of grab bars, slippery floor surface), kitchens (e.g., poor lighting, unsecured rugs), hallways, and staircases. The total home ergonomic risk factor score was then calculated as the sum of all positive (i.e., ‘yes’) responses on the checklist, with a higher aggregate score indicating a less safe home environment. The TUG test was administered by a trained physiotherapist, and participants were instructed to stand up from a chair, walk three meters, turn around, and return to the chair as quickly and safely as possible. Fall frequency was defined as the number of self-reported falls per participant over the two-month study period, with a fall defined as an event which resulted in a person coming to rest unintentionally on the ground or lower level.

The validity and reliability of the measurement tools were rigorously assessed to ensure the accuracy and consistency of the data collected. The Fear of Falling questionnaire, Berg Balance Scale, and Timed Up and Go test were evaluated using standard psychometric methods, including test-retest reliability and internal consistency analysis. McDonald's omega coefficient was employed to assess construct validity, yielding values above 0.85 for all instruments, indicating excellent reliability. These tools have been previously validated in Iranian studies on older populations, demonstrating satisfactory psychometric properties (Norouzi et al., 2023; Razmjouie et al., 2023).

### 2.4 Intervention

The four anti-slip flooring types (Sonia, Lotus, Aphrodite, Selda) were chosen based on a one-month market survey (February 2024) identifying them as the most common slip-resistant options in Iranian homes. While all were marketed as slip-resistant, they differed in texture and material; key surface roughness parameters (Ra, Rq, Rz) are reported in Supplementary Table 2.

The two-month intervention included:

1. **Home Safety Training and Modification:** Led by an occupational therapist and community health nurse, participants received personalized home assessments using a 96-item checklist, one-on-one education, and instructional materials with checklists and pictorial guides. Modifications focused on installing grab bars, applying non-slip mats, and improving lighting. Assistance with installation or equipment was provided as needed.
2. **Supervised Exercise Program:** Conducted twice weekly in groups of 10–12 at day care centers, each 60-min session was supervised by a physiotherapist and exercise instructor. Sessions included warm-up (10 min), strength training (20 min) targeting lower body muscles, balance exercises (20 min) such as single-leg stands and tandem walking, and cool-down stretching (10 min). Participants also received a booklet for safe home exercises on non-supervised days

### 2.5 Statistical analysis

Group means and standard deviations were calculated, and differences among the four flooring types were examined using ANOVA. Six pairwise comparisons were conducted with Bonferroni adjustment (α = 0.05/6 ≈ 0.008). Effect sizes were reported using partial eta squared (ηp^2^), omega squared (ω (World Health Organization, 2021)), and epsilon squared (ε (World Health Organization, 2021)) to provide complementary and increasingly unbiased estimates. Baseline demographic and health variables showed no significant differences based on the Kruskal–Wallis test; however, covariates such as age and chronic conditions were not controlled in the primary analysis. All analyses were performed in August 2024 using JAMOVI version 2.6.25.

### 2.6 Ethical considerations

Ethical standards were strictly followed. The study adhered to institutional and national guidelines, the 2013 Helsinki Declaration (with 2020 amendments), and frameworks such as STROBE (2009), ICMJE (2019), and the Belmont Report. Approval was granted by the Ethics Committee of the Nutrition and Food Industry Research Center, Shiraz University of Medical Sciences (IR.SUMS.SCHEANUT.REC.1401.142, March 15, 2024). Written informed consent was obtained, data were anonymized, confidentiality maintained, and participants could withdraw at any time without consequences. All procedures ensured safety, fairness, and equitable inclusion, and participants could access study findings upon request.

## 3 Results

### 3.1 Descriptive characteristics of participants

A total of 336 older women (84 per flooring group) from day care centers in Shiraz were included. Inclusion criteria were age 60–74, no history of injurious falls, Fear of Falling score ≥ 20, normal TUG results, and no reliance on assistive devices for daily mobility. Exclusion criteria included musculoskeletal problems, cognitive impairment, withdrawal, or death. Participants' mean age was 68.5 years (SD = 4.6); 80 % had at least primary education, 62 % reported average urban income, 70 % weighed 60–70 kg, and most lived in villas (95 %). Nearly all were married or widowed, with strong family support (87 % had ≥2 children). About 48 % reported daily walking for ∼1 h. While the exclusion criteria excluded full dependence on assistive devices, 10 participants (3 %) occasionally used a cane outdoors for confidence due to fear of falling. All participants could complete TUG and BBS tests without any device, confirming functional eligibility. Chronic conditions were present in 12 % (mostly controlled hypertension), 87 % reported high health satisfaction, 8 % had dizziness/imbalance, and 4.5 % reported minor falls. Data met ANOVA assumptions: normality (Kolmogorov-Smirnov, D'Agostino, Shapiro-Wilk, *p* = 0.1) and homogeneity of variances (Bartlett's and Levene's tests, *p* = 0.01). Kruskal-Wallis tests confirmed no baseline differences in demographic or health variables (*p* = 0.11).

### 3.2 Comparison of fear of falling before and after intervention

One-way ANOVA (Table 1) showed significant differences in fear of falling across flooring groups. Before the intervention, flooring type explained only ∼7.5 % of variance (partial η^2^ = 0.13; ω^2^ = 0.07). After the intervention, effect sizes increased (partial η^2^ = 0.39; ω^2^ = 0.34), indicating that the home safety and exercise program substantially reduced fear of falling, accounting for ∼34 % of the variance (*p* < 0.01). Post-hoc Bonferroni tests revealed that Sonia flooring led to greater reductions in fear of falling compared to Lotus, Aphrodite, and Selda (mean differences: Sonia–Lotus = 0.02, Sonia–Aphrodite = 0.04, Sonia–Selda = 0.06; *p* = 0.02). No significant differences were found among Lotus, Aphrodite, and Selda.

**Table 1:** Results of One-Way ANOVA Analysis for the Variable Fear of Falling (FoF) Before and After Using Flooring among Aged Iranian Women, 2025.

Dependent Variable	Source of Variation	Sum of Squares	df	Mean Square	F	p-value⁎	Partial Eta Squared	Epsilon Squared	Omega Squared
FOF Before Intervention	Between Groups	15.72	3	5.24	2.29	0.09	0.13	0.08	0.07
	Within Groups	100.75	81	2.29					
	Total	116.47	84						
FOF After Intervention	Between Groups	72.39	3	24.13	9.47	0.01	0.39	0.35	0.35
	Within Groups	112.08	81	2.54					
	Total	184.47	84						

⁎ *P*-value <0.05.

### 3.3 Comparison of balance (BBS-9) before and after the intervention

Table 2 shows that the intervention had minimal effect on balance. Before the intervention, flooring explained ∼2.8 % of variance (partial η^2^ = 0.09; ω^2^ = 0.03). After the intervention, effect sizes decreased (partial η^2^ = 0.02; ω^2^ = 0.04), and differences were not significant (*p* = 0.82). Bonferroni post-hoc comparisons revealed no meaningful differences between Sonia, Lotus, Aphrodite, and Selda (mean differences: 0.008–0.01; *p* > 0.05). The limited effect on balance and fall frequency may be due to the short two-month intervention, modest lifestyle changes, and resource constraints for home safety modifications. Longer interventions are recommended to observe measurable improvements in physical performance and fall risk.

**Table 2:** Results of one-way ANOVA Analysis for the variable berg balance before and after using flooring among aged Iranian Women, 2025.

Dependent Variable	Source of Variation	Sum of Squares	df	Mean Square	F	p-value⁎	Partial Eta Squared	Epsilon Squared	Omega Squared
Berg Before Intervention	Between Groups	12.39	3	4.13	1.45	0.23	0.09	0.02	0.02
	Within Groups	124.58	81	2.83					
	Total	136.97	84						
Berg After Intervention	Between Groups	9.41	3	3.13	0.3	0.82	0.02	0.04	0.046
	Within Groups	459.83	81	10.45					
	Total	469.25	84						

⁎ P-value <0.05.

### 3.4 Comparison of balance (TUG) before and after the intervention

As reported in Table 3, the one-way ANOVA results for the Timed Up and Go (TUG) test indicated that the intervention did not yield a statistically significant overall effect on mobility and dynamic balance. The pre-intervention effect sizes (partial η^2^ = 0.1, ω^2^ = 0.04) suggested that flooring type accounted for a small portion of the variance in TUG scores. Post-intervention, the non-significant *p*-value (*p* = 0.34) confirmed the lack of a statistically significant main effect. The observed effect size for the intervention was partial η^2^ = 0.07, which corresponds to a medium effect according to conventional benchmarks, though it was not statistically significant in this study.

**Table 3:** Results of One-Way ANOVA Analysis for the Variable TUG Balance Before and After Using Flooring among Aged Iranian Women, 2025.

Dependent Variable		Sum of Squares	df	Mean Square	F	p-value⁎	Partial Eta Squared	Epsilon Squared	Omega Squared
TUG Before Intervention	Between Groups	3.92	3	1.31	1.64	0.19	0.1	−0.04	−0.04
	Within Groups	35.05	81	0.79					
	Total	38.97	84						
TUG After Intervention	Between Groups	2.31	3	0.76	0.89	0.34	0.07	0.01	0.01
	Within Groups	29.38	81	0.66					
	Total	31.68	84						

⁎ P-value <0.05.

Despite the non-significant main effect, post-hoc pairwise comparisons (Bonferroni) revealed a specific pattern. Participants with Aphrodite flooring demonstrated statistically significant, albeit small, improvements in TUG scores compared to those with Sonia (mean difference = 0.03, *p* = 0.02), Lotus (mean difference = 0.04, p = 0.02), and Selda (mean difference = 0.05, *p* = 0.01) flooring. No other significant differences were observed between the other flooring types.

### 3.5 Comparison of fall frequency before and after the intervention

Table 4 shows that the intervention had minimal effect on fall frequency. Pre-intervention, flooring type explained only ∼3.2 % of variance (partial η^2^ = 0.03; ω^2^ = 0.03). Post-intervention, effect sizes remained small (partial η^2^ = 0.056; ω^2^ = 0.008), and overall differences were not significant (*p* = 0.46). Post-hoc tests indicated that Selda flooring led to modest but significant reductions in fall frequency compared to Sonia (0.02), Lotus (0.03), and Aphrodite (0.04) flooring (*p* = 0.02). No significant differences were observed among Sonia, Lotus, and Aphrodite.

**Table 4:** Results of One-Way ANOVA Analysis for the Variable Number of Falls Before and After Using Flooring among Aged Iranian Women, 2025.

Dependent Variable	Source of Variation	Sum of Squares	df	Mean Square	F	p-value⁎	Partial Eta Squared	Epsilon Squared	Omega Squared
Number of Falls Before Intervention	Between Groups	0.91	3	0.31	0.51	0.67	0.03	0.03	0.03
	Within Groups	26.33	81	0.59					
	Total	27.25	84						
Number of Falls After Intervention	Between Groups	2	3	0.66	0.89	0.46	0.05	0.01	0.01
	Within Groups	33.66	81	0.76					
	Total	35.66	84						

⁎ P-value <0.05.

### 3.6 Comparison of home ergonomic risk factors before and after the intervention

Table 5 shows that the intervention significantly reduced home ergonomic risk factors. Pre-intervention, flooring type explained ∼8.8 % of variance (partial η^2^ = 0.14; ω^2^ = 0.08). Post-intervention, effect sizes increased (partial η^2^ = 0.21; ω^2^ = 0.15), with overall differences being significant (*p* = 0.01). Bonferroni post-hoc tests indicated that Lotus flooring reduced ergonomic risks more effectively than Sonia (0.05), Aphrodite (0.07), and Selda (0.08) flooring. No significant differences were observed among Sonia, Aphrodite, and Selda.

**Table 5:** Results of One-Way ANOVA Analysis for the Variable Risk Factors Before and After Using Flooring among Aged Iranian Women, 2025.

Dependent Variable	Source of Variation	Sum of Squares	df	Mean Square	F	p-value⁎	Partial Eta Squared	Epsilon Squared	Omega Squared
Risk Factors Before Intervention	Between Groups	165.83	3	55.27	2.53	0.07	0.14	0.08	0.08
	Within Groups	959.16	81	21.79					
	Total	1125	84						
Risk Factors After Intervention	Between Groups	316.39	3	105.46	3.89	0.01	0.21	0.15	0.15
	Within Groups	1191.08	81	27.1					
	Total	1507.47	84						

⁎ P-value <0.05.

Supplementary Table 3 presents descriptive statistics and Bonferroni post-hoc comparisons for fear of falling, balance (BBS and TUG), fall frequency, and home ergonomic risk. The intervention significantly reduced fear of falling, particularly in the Sonia flooring group, with mean scores decreasing from 25.3 (SD = 4.2) to 18.7 (SD = 3.8). Pairwise comparisons showed significant differences versus Lotus (−6.6, *p* = 0.01), Aphrodite (−7.5, *p* = 0.02), and Selda (−8.3, p = 0.02). The partial η^2^ of 0.39 indicates that the intervention explained ∼39 % of the variance in fear of falling. Balance measures (BBS and TUG) showed minimal changes. For example, BBS in the Sonia group changed from 45.2 (SD = 3.1) to 45 (SD = 3), and TUG decreased from 12.3 (SD = 1.5) to 12.2 (SD = 1.4), with small effect sizes (partial η^2^ = 0.02 for BBS; 0.07 for TUG), indicating limited impact.

Home ergonomic risks improved significantly, especially for the Lotus flooring group. Mean scores dropped from 36 (SD = 5.3) to 28.5 (SD = 4.6), with pairwise differences of −7.5 versus Aphrodite (p = 0.01) and − 7.6 versus Selda (p = 0.01). The partial η^2^ of 0.21 shows ∼21 % of variance explained. In summary, tailored interventions combining home safety training and exercises effectively reduced fear of falling and improved home safety, particularly for Sonia (fear of falling) and Lotus (ergonomic risk) flooring groups, while balance outcomes remained largely unchanged.

## 4 Discussion

The intervention significantly reduced fear of falling among older adults, underscoring the influence of psychological factors on functional performance and daily activity—findings consistent with prior research showing fear of falling negatively affects quality of life (Stanghelle et al., 2020; Lytras et al., 2022). However, no significant improvements were detected in balance measures (BBS-9, TUG), which may reflect limited lifestyle changes and financial barriers to implementing home modifications. This differs from studies that reported balance gains following exercise-based programs (Huang et al., 2022; Donatoni da Silva et al., 2022; Di Lorito et al., 2021).

Fall frequency also showed no significant change, contrasting with evidence that exercise or increased awareness can reduce falls (Liu-Ambrose et al., 2019; Flint et al., 2020). In some cases, heightened awareness may temporarily increase anxiety (Kuhirunyaratn et al., 2019). Nevertheless, home-safety training led to a 15 % improvement in ergonomic conditions, particularly in high-risk areas such as bathrooms and kitchens. These findings align with studies highlighting the preventive value of environmental modifications (Boonkhao et al., 2024; La Porta et al., 2022; Alves et al., 2024; Gerards et al., 2023).

Despite frequent prior falls, participants had limited knowledge of basic prevention strategies, and nearly 60 % of falls occurred indoors without subsequent corrective changes—emphasizing the need for targeted education and practical guidance for older adults.

Several limitations warrant consideration. The two-month duration, constrained by the thesis timeline, may have been insufficient to yield measurable changes in balance or fall frequency; longer follow-up periods are needed. The study included only older women, limiting generalizability, although this focus is justified given women's greater exposure to domestic hazards, higher musculoskeletal vulnerability, and longer life expectancy. The hot, dry climate of southern Iran may also have influenced indoor exposure and fall risk. Furthermore, although baseline demographics were similar across flooring groups, potential confounders (e.g., age, chronic conditions) were not statistically adjusted; future studies with larger samples could incorporate ANCOVA to better estimate intervention effects.

Despite these constraints, the findings have practical implications. Reductions in fear of falling and ergonomic risk factors reflect the value of combining home-safety education, environmental modifications, and structured movement exercises. Simple measures—non-slip flooring, grab bars, and improved lighting—can reduce hazards, and culturally tailored programs that consider older women's household roles may enhance uptake. These strategies can be integrated into community services and public health initiatives and may benefit from supportive policies or subsidies.

Future studies should use longer intervention periods (6–12 months), include both genders, and involve multiple centers across diverse climates and seasons to better capture environmental influences. Advanced analytic approaches (e.g., SEM) may help identify pathways linking psychological factors, ergonomic changes, and fall outcomes. Continued attention to confidence and anxiety reduction is also needed, given their central role in fall prevention.

## 5 Conclusion

Home safety training and ergonomic modifications effectively reduced fear of falling and improved home safety among older women. However, longer and multi-component interventions are needed to enhance balance and reduce fall frequency. Integrating physical and psychological strategies into fall prevention programs can help create safer environments and promote healthy aging.

## Human & animal rights

No animals were used for studies that are the basis of this research. This research was conducted on humans are in accordance with the Helsinki Declaration of 1975, as revised in 2013 (http://ethics.iit.edu/ecodes/node/3931).

## The standard for reporting

The STROBE guidelines and methodology as well as the 2013 Helsinki Declaration (including its 2020 amendments), and ICMJE (2019), and the principles outlined in the Belmont Report was followed during this study. All procedures conducted in this study involving human participants were in compliance with the ethical standards of the institutional and national research committees and its subsequent amendments, which emphasize informed consent and the confidentiality of personal information.

## CRediT authorship contribution statement

**Saiedeh Nazari:** Writing – review & editing, Writing – original draft, Software, Resources, Investigation, Data curation, Conceptualization. **Fakhruddin Boraghi:** Writing – review & editing, Writing – original draft, Visualization, Validation, Methodology, Investigation, Conceptualization. **Masoud Karimi:** Writing – review & editing, Writing – original draft, Project administration, Methodology, Investigation. **Mojtaba Kamalinia:** Writing – review & editing, Writing – original draft, Visualization, Validation, Software, Resources, Project administration, Methodology, Investigation. **Samuel T. Faloye:** Writing – review & editing, Writing – original draft, Formal analysis, Data curation, Conceptualization. **Abdolrahim Asadollahi:** Writing – review & editing, Writing – original draft, Validation, Supervision, Software, Formal analysis, Conceptualization.

## Patient Consent

Written and verbal consent of a sample was obtained before participating the study.

## Consent for publication

Not applicable.

## Ethical considerations

Ethical approval was obtained from the Ethics Committee of Shiraz University of Medical Sciences (Code: IR.SUMS.SCHEANUT.REC.1401.142). Written informed consent was secured from all participants, ensuring their data remained confidential and used solely for research purposes. Participants were informed of the study's objectives and could withdraw at any stage without consequences. The study adhered to ethical standards, including the Helsinki Declaration, STROBE, and Belmont Report principles, while minimizing risks and maintaining participant privacy.

## Declaration of generative AI and AI-assisted Technologies in the Writing Process

During the preparation of this work, the authors did not use generative AI or AI-assisted technologies for study design, data analysis, or the creation of textual/visual content (including figures, tables, and captions). Artificial intelligence tools were employed solely for grammar and language editing (e.g., QWEN-MAX V. 2.5 and Grammarly) to improve readability.

## Funding

None.

## Declaration of competing interest

The authors declare the following financial interests/personal relationships which may be considered as potential competing interests: Abdolrahim Asadollahi reports administrative support was provided by Shiraz University of Medical Sciences. If there are other authors, they declare that they have no known competing financial interests or personal relationships that could have appeared to influence the work reported in this paper.

## Data Availability

Data will be made available on request.

## Appendix A Supplementary data

Collection of Online Supplementary Tables 1, 2 & 3. Image 1
