# Supplementary material for: Effectiveness of home safety training and balance exercises in reducing fear of falling among older women: A quasi-experimental study in southern Iran
Source: Prev Med Rep. 2025 Dec 15;61:103351. doi: 10.1016/j.pmedr.2025.103351 (PMC12771351; doi:10.1016/j.pmedr.2025.103351)
Supplement: Supplementary file 1 — Collection of Online Supplementary Tables 1, 2 & 3. [file mmc1.docx]

**Collection of Online Supplementary Tables**

**Online Supplementary Table 1 Baseline Demographic and Clinical Characteristics of Participants by Flooring Type among Aged Iranian Women, 2025**

| **Characteristic** | **Overall (N=48)** | **Sonia (n=12)** | **Lotus (n=12)** | **Aphrodite (n=12)** | **Selda (n=12)** | **p-value** |
| --- | --- | --- | --- | --- | --- | --- |
| **Age (years), Mean (SD)** | 68.5 (4.6) | 67.8 (4.9) | 69.1 (4.2) | 68.3 (4.8) | 68.7 (4.5) | 0.91 |
| **Education Level, n (%)** |  |  |  |  |  | 0.33 |
| Illiterate | 14 (29.2%) | 2 (16.7%) | 2 (16.7%) | 5 (41.7%) | 5 (41.7%) |  |
| Primary School | 18 (37.5%) | 5 (41.7%) | 4 (33.3%) | 4 (33.3%) | 5 (41.7%) |  |
| Middle School | 12 (25.0%) | 4 (33.3%) | 6 (50%) | 0 (0.0%) | 2 (16.7%) |  |
| High School Diploma | 4 (8.3%) | 1 (8.3%) | 0 (0.0%) | 3 (25%) | 0 (0.0%) |  |
| **Marital Status, n (%)** |  |  |  |  |  | 0.43 |
| Married | 23 (47.9%) | 7 (58.3%) | 6 (50%) | 4 (33.3%) | 6 (50%) |  |
| Widowed | 15 (31.3%) | 4 (33.3%) | 4 (33.3%) | 6 (50%) | 1 (8.3%) |  |
| Living with Others | 9 (18.8%) | 1 (8.3%) | 2 (16.7%) | 2 (16.7%) | 4 (33.3%) |  |
| Divorced | 1 (2.1%) | 0 (0.0%) | 0 (0.0%) | 1 (8.3%) | 0 (0%) |  |
| **Chronic Conditions, n (%)** |  |  |  |  |  |  |
| Metabolic Syndrome | 17 (35.4%) | 7 (58.3%) | 3 (25.0%) | 3 (25.0%) | 4 (33.3%) | 0.46 |
| Heart Disease | 10 (20.8%) | 2 (16.7%) | 2 (16.7%) | 3 (25.0%) | 3 (25%) |  |
| Diabetes | 9 (18.8%) | 2 (16.7%) | 1 (8.3%) | 3 (25.0%) | 3 (25%) |  |
| Arthritis | 6 (12.5%) | 0 (0.0%) | 3 (25.0%) | 3 (25.0%) | 0 (0.0%) |  |
| Osteoporosis | 4 (8.3%) | 1 (8.3%) | 2 (16.7%) | 0 (0.0%) | 1 (8.3%) |  |
| Depression | 2 (4.2%) | 0 (0.0%) | 1 (8.3%) | 0 (0.0%) | 1 (8.3%) |  |
| **Health Satisfaction, n (%)** |  |  |  |  |  | 0.41 |
| Satisfied | 25 (52.1%) | 8 (66.7%) | 4 (33.3%) | 7 (58.3%) | 6 (50%) |  |
| Not Satisfied | 23 (47.9%) | 4 (33.3%) | 8 (66.7%) | 5 (41.7%) | 6 (50%) |  |
| **Medication Use, n (%)** | 40 (83.3%) | 10 (83.3%) | 9 (75.0%) | 10 (83.3%) | 11 (91.7%) | 0.75 |
| **Financial Support, n (%)** | 24 (50.0%) | 6 (50%) | 8 (66.7%) | 4 (33.3%) | 6 (50%) | 0.45 |
| **Dizziness while Walking, n (%)** | 24 (50.0%) | 4 (33.3%) | 6 (50%) | 6 (50%) | 8 (66.7%) | 0.37 |
| **History of Falls, n (%)** | 22 (45.8%) | 5 (41.7%) | 7 (58.3%) | 4 (33.3%) | 6 (50.0%) | 0.65 |
| **Type of Housing, n (%)** |  |  |  |  |  | 0.52 |
| Apartment | 27 (56.3%) | 6 (50%) | 8 (66.7%) | 5 (41.7%) | 8 (66.7%) |  |
| Villa | 21 (43.8%) | 6 (50%) | 4 (33.3%) | 7 (58.3%) | 4 (33.3%) |  |
| ***Note****: Data are presented as Mean (Standard Deviation) for age and as Frequency (Percentage) for categorical variables.*  *P-values were derived from One-Way ANOVA for age and from Kruskal-Wallis tests for categorical variables to assess baseline homogeneity between the four flooring groups.*  *The total N for the study was 336; this table presents a detailed breakdown for a representative subset (n=48) that was used for in-depth demographic analysis. * P-value < 0.05.* | | | | | | |

-----------------------

**Online Supplementary Table 2. Physical and Chemical Characteristics of the Selected Anti-Slip Flooring Types**

| **Floor**  **Covering** | **Surface Roughness**  **(Ra, µm)** | **Root Mean Square Roughness**  **(Rq, µm)** | **Maximum Height of Profile**  **(Rz, µm)** | **Prevalence in Market**  **(%)** | **Manufacturer Safety**  **Rating** |
| --- | --- | --- | --- | --- | --- |
| Aphrodite | 2.5 | 3 | 15 | 30 | High |
| Lotus | 1.8 | 2.2 | 12.5 | 25 | Medium-High |
| Selda | 3.2 | 3.8 | 18 | 20 | High |
| Sonia | 2 | 2.5 | 14 | 25 | Medium |
| ***Note****: Surface roughness parameters (Ra, Rq, Rz) were obtained from manufacturers’ technical catalogs.*  *Prevalence in the market is based on sales data from local distributors.* | | | | | |

-------------------

**Online Supplementary Table 3: Descriptive Statistics and Post-hoc Test Results for Fear of Falling, Berg Balance, TUG, Number of Falls, and Risk Factors among Aged Iranian Women, 2025**

| **Variable** | **Group** | **Pre-Intervention (Mean, SD)** | **Post-Intervention (Mean, SD)** | **Effect Size (Partial Eta²)** | **p-value** | **Post-hoc Comparisons: Mean Diff** |
| --- | --- | --- | --- | --- | --- | --- |
| Fear of Falling | Sonia | 25.30, 4.20 | 18.70, 3.8 | 0.392 | 0.01 | -6.60 (vs Lotus, p < 0.05), -7.50 (vs Aphrodite, p < 0.05), -8.30 (vs Selda, p < 0.05) |
|  | Lotus | 26.10, 4.50 | 20.40, 4.1 | 0.392 | 0.02 | -5.70 (vs Aphrodite, p < 0.05), -6.50 (vs Selda, p < 0.05) |
|  | Aphrodite | 25.80, 4.30 | 21.20, 4 | 0.392 | 0.03 | -4.80 (vs Selda, p < 0.05) |
|  | Selda | 26.00, 4.40 | 20.90, 4.2 | 0.392 | 0.01 | -5.1 |
| Berg Balance | Sonia | 45.20, 3.10 | 45.00, 3 | 0.02 | 0.82 | -0.20 (vs Lotus, p > 0.05), -0.30 (vs Aphrodite, p > 0.05), -0.40 (vs Selda, p > 0.05) |
|  | Lotus | 44.80, 3.20 | 44.70, 3.1 | 0.02 | 0.79 | -0.10 (vs Aphrodite, p > 0.05), -0.20 (vs Selda, p > 0.05) |
|  | Aphrodite | 45.00, 3.00 | 44.90, 3 | 0.02 | 0.86 | -0.10 (vs Selda, p > 0.05) |
|  | Selda | 44.90, 3.10 | 44.80, 3 | 0.02 | 0.90 | -0.1 |
| TUG Balance | Sonia | 12.30, 1.50 | 12.20, 1.4 | 0.07 | 0.47 | -0.10 (vs Lotus, p > 0.05), -0.20 (vs Aphrodite, p > 0.05), -0.30 (vs Selda, p > 0.05) |
|  | Lotus | 12.50, 1.60 | 12.40, 1.5 | 0.07 | 0.51 | -0.10 (vs Aphrodite, p > 0.05), -0.20 (vs Selda, p > 0.05) |
|  | Aphrodite | 12.40, 1.50 | 12.30, 1.4 | 0.07 | 0.63 | -0.10 (vs Selda, p > 0.05) |
|  | Selda | 12.60, 1.60 | 12.50, 1.5 | 0.07 | 0.72 | -0.1 |
| Fall Frequency | Sonia | 1.80, 0.70 | 1.70, 0.6 | 0.05 | 0.61 | -0.10 (vs Lotus, p > 0.05), -0.20 (vs Aphrodite, p > 0.05), -0.30 (vs Selda, p > 0.05) |
|  | Lotus | 1.90, 0.80 | 1.80, 0.7 | 0.05 | 0.55 | -0.10 (vs Aphrodite, p > 0.05), -0.20 (vs Selda, p > 0.05) |
|  | Aphrodite | 1.80, 0.70 | 1.70, 0.6 | 0.05 | 0.69 | -0.10 (vs Selda, p > 0.05) |
|  | Selda | 1.70, 0.60 | 1.60, 0.5 | 0.05 | 0.78 | -0.1 |
| Home Ergonomic Risk | Sonia | 35.20, 5.10 | 30.10, 4.8 | 0.21 | 0.01 | -5.10 (vs Lotus, p < 0.05), -5.20 (vs Aphrodite, p < 0.05), -5.30 (vs Selda, p < 0.05) |
|  | Lotus | 36.00, 5.30 | 28.50, 4.6 | 0.21 | 0.01 | -7.50 (vs Aphrodite, p < 0.05), -7.60 (vs Selda, p < 0.05) |
|  | Aphrodite | 35.80, 5.20 | 29.80, 4.7 | 0.21 | 0.04 | -6.00 (vs Selda, p < 0.05) |
|  | Selda | 35.50, 5.00 | 29.50, 4.5 | 0.21 | 0.02 | -6.0 |
| ** P-value < 0.05.* | | | | | | |
